# Mechanical and Electronic Properties of XC_6_ and XC_12_

**DOI:** 10.3390/ma9090726

**Published:** 2016-08-25

**Authors:** Qun Wei, Quan Zhang, Meiguang Zhang

**Affiliations:** 1 School of Physics and Optoelectronic Engineering, Xidian University, Xi’an 710071, China; 2 School of Microelectronics, Xidian University, Xi’an 710071, China; quzhang93@foxmail.com; 3 College of Physics and Optoelectronic Technology, Baoji University of Arts and Sciences, Baoji 721016, China

**Keywords:** first-principles calculations, carbides, mechanical properties, elastic anisotropy

## Abstract

A series of carbon-based superconductors XC_6_ with high *T_c_* were reported recently. In this paper, based on the first-principles calculations, we studied the mechanical properties of these structures, and further explored the XC_12_ phases, where the X atoms are from elemental hydrogen to calcium, except noble gas atoms. The mechanically- and dynamically-stable structures include HC_6_, NC_6_, and SC_6_ in XC_6_ phases, and BC_12_, CC_12_, PC_12_, SC_12_, ClC_12_, and KC_12_ in XC_12_ phases. The doping leads to a weakening in mechanical properties and an increase in the elastic anisotropy. C_6_ has the lowest elastic anisotropy, and the anisotropy increases with the atomic number of doping atoms for both XC_6_ and XC_12_. Furthermore, the acoustic velocities, Debye temperatures, and the electronic properties are also studied.

## 1. Introduction

Elemental carbon exhibits a rich diversity of structures and properties, due to its flexible bond hybridization. A large number of stable or metastable phases of the pure carbon, including the most commonly known, graphite and diamond, and other various carbon allotropes [1,2,3,4] (such as lonsdaleite, fullerene, and graphene, etc.), and diversified carbides [5,6,7,8,9,10,11], have been studied in experiments and theoretical calculations. Graphite, which is the most stable phase at low pressure, has a *sp*^2^-hybridized framework and is ultrasoft semimetallic, whereas diamond, stable at high pressure, is superhard, insulating with a *sp*^3^ network. Recently, a novel one-dimensional metastable allotrope of carbon with a finite length was first synthesized by Pan et al. [1], called Carbyne. It has a *sp*-hybridized network and shows a strong purple-blue fluorescence. The successful synthesis of Carbyne is a great promotion for the further analysis on properties and applications. The 2D material MXenes as a promising electrode material, which is early transition metal carbides and carbon nitrides, is reported [11], owing to its metallic conductivity and hydrophilic nature. These properties of different carbides are appealing. To find superhard superconductors, researches designed some carbide superconductors, such as boron carbides and XC_6_ structure with cubic symmetry. The diamond-like B_x_C_y_ system, which is superhard and superconductive, has also attracted much interest [5,6,7,8,9,10]. The best simulated structure of the synthesized d-BC_3_ (Pmma-b phase) has a Vickers hardness of 64.8 GPa, showing a superhard nature, and its *T_c_* reaches 4.9–8.8 K [5]. The P-4m2 polymorph of d-BC_7_ with a low energy also has a high Vickers hardness of 75.2 GPa [8]. Furthermore, Wang et al. [9] explored more potential superhard structures of boron carbide, uncovering the stability is mainly contributed by the elemental boron at low pressure, and by the carbon at high pressure. The novel metastable carbon structure C_6_ bcc is predicted with a cubic symmetry [12]. It is an indirect band gap semiconductor with 2.5 eV, calculated by the local density approximation. Recently, doped with simple metals, Lu et al. [13] studied a series of sodalite-based carbon structures, similar to the boron-doped diamond. Although they found these structures are all metastable, some of these structures show a superconductivity, e.g., the critical temperature of NaC_6_ is 116 K. In this paper, we mainly study the mechanical properties of these eleven XC_6_ phases (HC_6_, LiC_6_, NC_6_, OC_6_, FC_6_, NaC_6_, AlC_6_, SiC_6_, PC_6_, SC_6_, and ClC_6_) which is of dynamical stability and, for comparison, C_6_ is also calculated. In addition, the XC_12_ structures are systematically explored, in which the X atom is from H to Ca, except He, Ne, and Ar. The doping-induced changes in elastic constant, modulus, the anisotropy of elasticity and acoustic velocity, Debye temperature, and the electronic structures are also studied.

## 2. Results and Discussion

**Figure 1:**
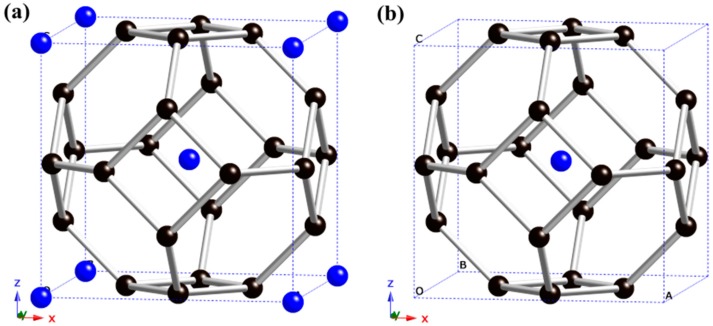
Unit cell of XC_6_ (**a**) and XC_12_ (**b**). The black and blue spheres represent C and X atoms, respectively.

As shown in Figure 1a, the structure of XC_6_ is obtained by doping the X atom into the C_6_ bcc structure at (0, 0, 0). It is of Im-3m symmetry (No. 229), consisting of two formula units (f.u.) per unit cell. Each C atom has four nearest neighbors with the bond angle of 90° or 120°. The XC_6_ structure has four C_4_ rings and eight C_6_ rings. In Table 1, the calculated lattice parameter *a* of C_6_ has a good agreement with the available result [12], and is smaller than that of the XC_6_ structures. By removing the corner atoms and only leaving the center X atom, the XC_12_ structure is obtained (Figure 1b). All of the XC_12_ phases are smaller than the corresponding XC_6_ phases, but larger than the C_6_ phase in the lattice parameter. 

**Figure 2:**
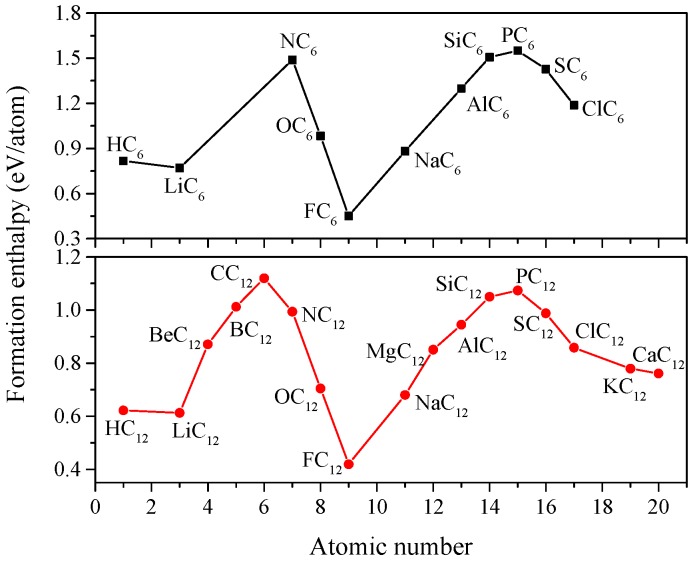
Formation enthalpy of XC_6_ and XC_12_.

The formation enthalpies of XC_6_ in [13] and XC_12_ structures are calculated reference to diamond and the most stable X phase at ambient pressure. The equations are given by $\Delta H_{XC_{6}}=\left(H_{XC_{6}}-H_{X}-6H_{C}\right)/7$, and $\Delta H_{XC_{12}}=\left(H_{XC_{12}}-H_{X}-12H_{C}\right)/13$, and the calculated results are shown in Figure 2. The positive values indicate these phases are metastable. The two curves of the formation enthalpy follow a similar trend, where the F-doped carbides have the lowest Δ*H*, and the PC_6_ and CC_12_ have the largest Δ*H* in XC_6_ and XC_12_, respectively. Compared to other doped elements of the second and the third periods in the XC_6_ and XC_12_, fluorine (F) possesses the largest electronegativity difference relative to C, leading to a stronger interaction between F and C atoms; thus, FC_6_ and FC_12_ phases are more stable.

**Figure 3:**
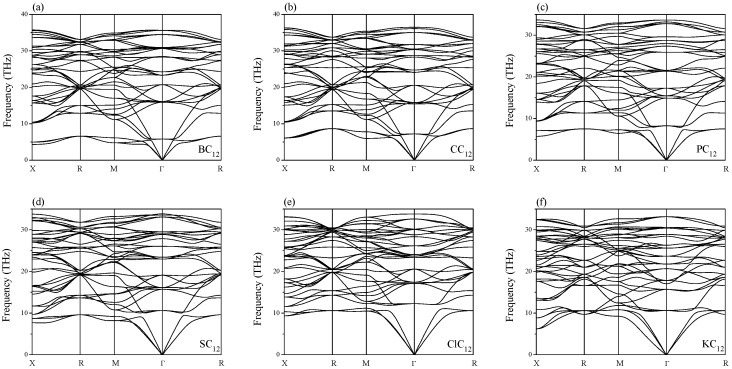
Phonon spectra of dynamically stable phases (**a**) BC_12_; (**b**) CC_12_; (**c**) PC_12_; (**d**) SC_12_; (**e**) ClC_12_; and (**f**) KC_12_.

**Figure 4:**
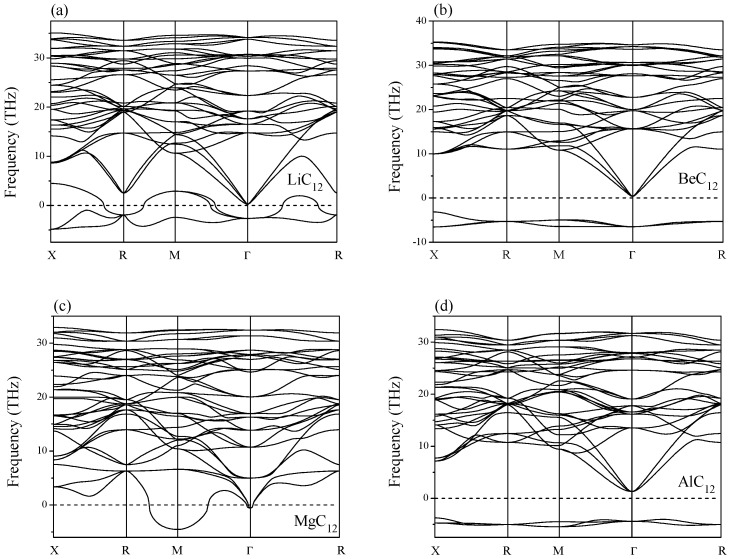
Phonon spectra of dynamically unstable phases (**a**) LiC_12_; (**b**) BeC_12_; (**c**) MgC_12_; and (**d**) AlC_12_.

The calculated elastic constants and moduli are listed in Table 1. The generalized Born’s mechanical stability criteria of cubic phase are given by [15]: $C_{11}>0,C_{44}>0,\;C_{11}>\left|C_{12}\right|,$ and $(C_{11}+2C_{12})>0.$ In Table 1, the C_6_ and HC_6_, NC_6_, and SC_6_ have the mechanical stability, and they are also dynamically stable [13]. The XC_12_ has ten mechanically stable phases, but only six of these phases have the dynamical stability (BC_12_, CC_12_, PC_12_, SC_12_, ClC_12_, and KC_12_) due to the absence of the imaginary frequency in the whole Brillouin zone (see Figure 3 and Figure 4). The S is the only element that is capable to make not only XC_6_, but also XC_12_, stable.

By Voigt-Reuss-Hill approximations [16,17,18], the bulk modulus *B* and shear modulus *G* can be obtained, and the Young’s modulus *E* and Poisson’s ratio ν are defined as [19,20] E=9BG/(3B+G) and ν=(3B−2G)/[2(3B+G)]. HC_6_ has the largest bulk modulus of 346 GPa, showing the best ability to resist the compression. The shear modulus is often used to qualitatively predict the hardness, and Young’s modulus *E* is defined as the ratio between stress and strain to measure the stiffness of a solid material. In Table 1, C_6_ is the largest in shear modulus and Young’s modulus, which means that doping leads to a weakening in mechanical properties. The Poisson’s ratio exhibits the plasticity; usually, the larger the value, the better the plasticity. According to Pugh [21], C_6_, HC_6_, BC_12_, CC_12_, and PC_12_ are brittle materials (*B*/*G* < 1.75), while NC_6_, SC_6_, SC_12_, ClC_12_, and KC_12_ are ductile materials (*B*/*G* > 1.75). This conforms the calculated results of Poisson’s ratio.

The elastic anisotropy is important for the analysis on the mechanical property and, thus, the universal elastic anisotropy index (*A^U^*), Zener anisotropy index (*A*), and the percentage anisotropy in compressibility and shear are calculated. For the cubic phase, the universal elastic anisotropy index [22] is defined as: $A^{U}=5G_{V}/G_{R}+B_{V}/B_{R}-6$, the nonzero value suggests an anisotropy characteristic. Furthermore, it is known that *C*_44_ represents the resistance to deformation with respect to a shear stress applied across the (100) plane in the [010] direction, and $(C_{11}-C_{12})/2$ represents the resistance to shear deformation by a shear stress applied across the (110) plane in the $\left[1\bar{1}0\right]$ direction. For an isotropic crystal, the two shear resistances turn to identical. Therefore, Zener [23] introduced $A=2C_{44}/(C_{11}-C_{12})$ to quantify the extension of anisotropy. The value of 1.0 represents the isotropy, and any deviation from 1.0 indicates the degree of the shear anisotropy. The percentage anisotropy in compressibility and shear are given by: $A_{B}=(B_{V}-B_{R})/\left(B_{V}+B_{R}\right)$ and $A_{G}=\left(G_{V}-G_{R}\right)/\left(G_{V}+G_{R}\right)$ [24]. The *A_B_* is always 0.0 for a cubic phase. As shown in Table 2, C_6_ has the lowest anisotropy. The universal elastic anisotropy index and the percentage anisotropy in shear is increasing with the atomic number of doped element for both XC_6_ and XC_12_, and the anisotropy which obtains from the shear anisotropic factor is also increasing, except SC_6_ and KC_12_. Furthermore, owing to the percentage anisotropy in shear of C_6_, BC_12_, and CC_12_ being slight, they are almost isotropic.

**Figure 5:**
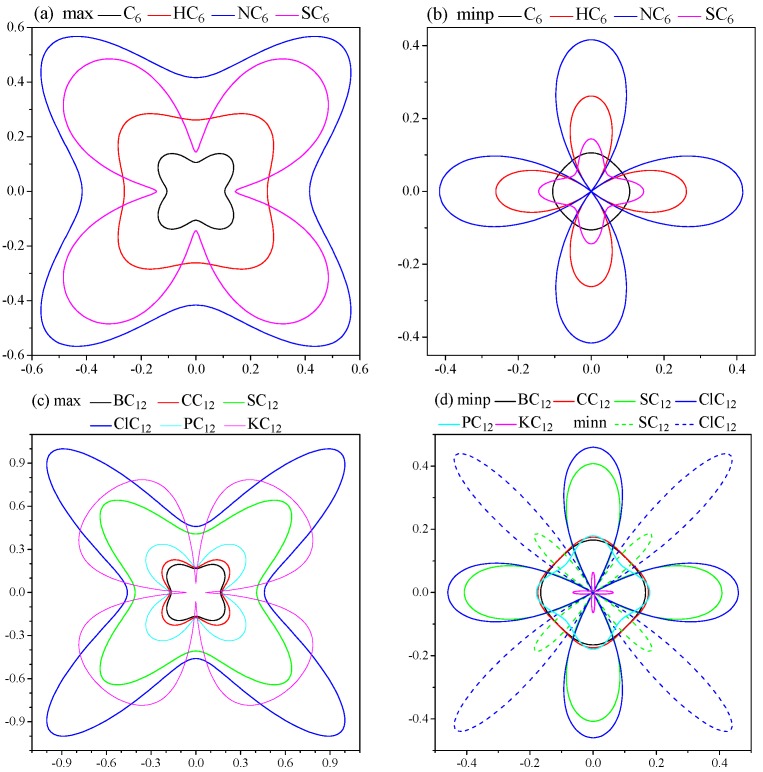
2D representations of Poisson’s ratio. (**a**) Maximum of C_6_ and XC_6_ stable phases; (**b**) minimum positive of C_6_ and XC_6_ stable phases; (**c**) maximum of XC_12_ stable phases; and (**d**) minimum positive and minimum negative of XC_12_ stable phases; particularly, only SC_12_ and ClC_12_ have the negative minimum Poisson’s ratio, the solid and dash lines represent the minimum positive and minimal negative, respectively.

The elastic anisotropies are calculated with the elastics anisotropy measures (ElAM) code [25,26] which makes the representations of non-isotropic materials easy and visual. For the cubic phase, the representation in *xy*, *xz*, and *yz* planes are identical, as a result, only the *xy* plane is presented. The 2D figures of the differences in each direction of Poisson’s ratio are shown in Figure 5. The maximum value curves and minimum positive value curves of C_6_ and XC_6_ stable phases are illustrated in Figure 5a,b, and those of XC_12_ stable phases are shown in Figure 5c,d. Particularly, the SC_12_ and ClC_12_ have the negative minimum Poisson’s ratio. It is seen that all of the structures are anisotropic and C_6_ has the lowest anisotropy, suggesting the doping increase the elastic anisotropy. The largest value of maximum curve is in the same direction of the lowest value of minimum positive value curve for each structure. Furthermore, for XC_12_ phases, the anisotropy of Poisson’s ratio is increasing with the atomic number. The negative minimum Poisson’s ratio of SC_12_ and ClC_12_ indicate these two phases have auxeticity [27], and ClC_12_ is more prominent than SC_12_.

**Figure 6:**
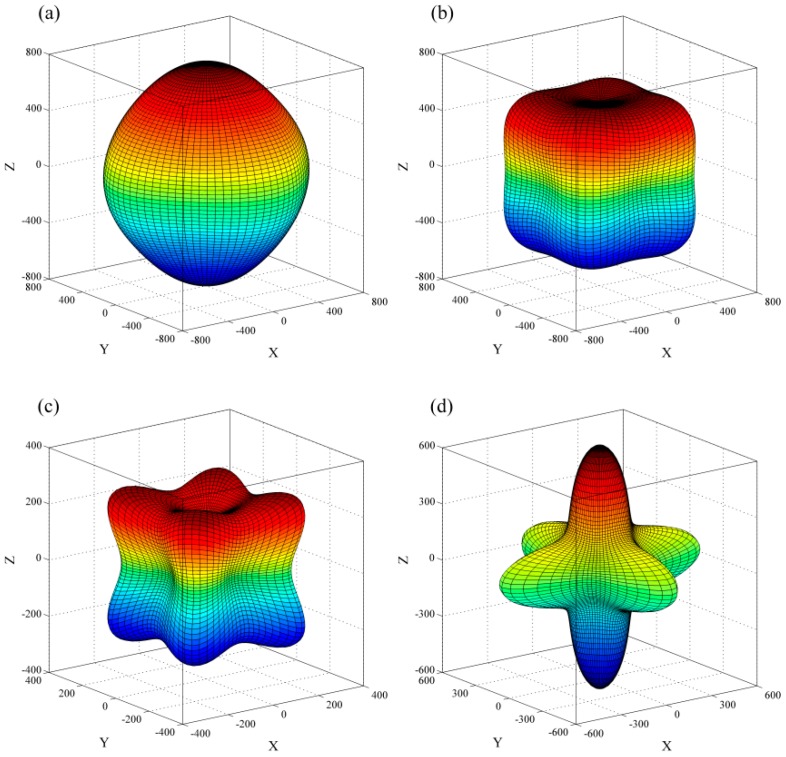
Directional dependence of the Young’s modulus of C_6_ (**a**); HC_6_ (**b**); NC_6_ (**c**); and SC_6_ (**d**).

**Figure 7:**
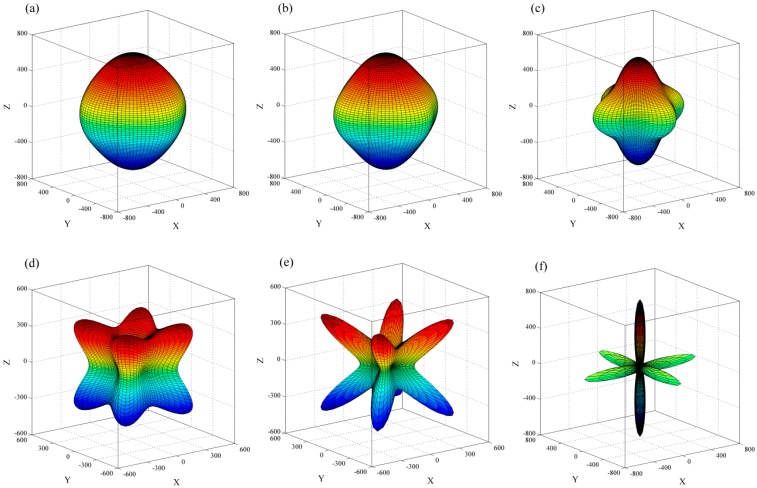
Directional dependence of the Young’s modulus of BC_12_ (**a**); CC_12_ (**b**); PC_12_ (**c**); SC_12_ (**d**); ClC_12_ (**e**); and KC_12_ (**f**).

The directional dependence of the Young’s modulus [28] are demonstrated in Figure 6 and Figure 7. The distance from the origin of system of coordinate to the surface equals the Young’s modulus in this direction, and thus any departure from the sphere indicates the anisotropy. As shown, all of the phases are anisotropic, and the anisotropy of Young’s modulus is increasing with the doping atomic number. For the S-doped phases, which have stable XC_6_ and XC_12_ structures, the maximum (minimum) values of SC_6_ and SC_12_ are 650 (291) and 371 (175) GPa, respectively. The *E*_max_/*E*_min_ ratio of SC_6_ (2.23) is slightly larger than that of SC_12_ (2.12), indicating the SC_6_ is more anisotropic.

The acoustic velocity is a fundamental parameter to measure the chemical bonding characteristics, and it is determined by the symmetry of the crystal and propagation direction. Brugger [29] provided an efficient procedure to calculate the phase velocities of pure transverse and longitudinal modes from the single crystal elastic constants. The cubic structure only has three directions [001], [110], and [111] for the pure transverse and longitudinal modes and other directions are for the qusi-transverse and qusi-longitudinal waves. The acoustic velocities of a cubic phase in the principal directions are [30]:

for [100], $v_{l}=\sqrt{C_{11}/\rho },\;[010]v_{t1}=[001]v_{t2}=\sqrt{C_{44}/\rho },$
for [110], $v_{l}=\sqrt{\left(C_{11}+C_{12}+2C_{44}\right)/2\rho },\;[1\bar{1}0]v_{t1}=\sqrt{\left(C_{11}-C_{12}\right)/2\rho },\;[001]v_{t2}=\sqrt{C_{44}/\rho },$
for [111], $v_{l}=\sqrt{\left(C_{11}+2C_{12}+4C_{44}\right)/3\rho },\;[11\bar{2}]v_{t1}=v_{t2}=\sqrt{\left(C_{11}-C_{12}+C_{44}\right)/3\rho }.$

where ρ is the density of the structure, *v_l_* is the longitudinal acoustic velocity, and *v_t_*_1_ and *v_t_*_2_ refer the first transverse mode and the second transverse mode, respectively. It should be noted that there is a misprint for equation of $[1\bar{1}0]v_{t1}$ in [30]. Here, the correct expression is given. Based on the elastic constants, the anisotropic properties of acoustic velocities indicate the elastic anisotropy in these crystals. As a fundamental physical parameter which correlates with many physical properties of solids, the Debye temperature can be obtained from the average acoustic velocity: $\Theta_{D}=\frac{h}{k_{B}}{\left[\frac{3n}{4\pi }\left(\frac{N_{A}\rho }{M}\right)\right]}^{1/3}v_{m}$, where *h* and *k_B_* are the Planck and Boltzmann constants, respectively; *N_A_* is Avogadro’s number; *n* is the total number of atoms in the formula unit; *M* is the mean molecular weight, and ρ is the density. The average acoustic velocity is $v_{m}={\left[(2/v_{tm}^{3}+1/v_{lm}^{3})/3\right]}^{-1/3}$, where $v_{lm}=\sqrt{(B+4G/3)/\rho }$ is the average longitudinal acoustic velocity, and $v_{tm}=\sqrt{G/\rho }$ is the average transverse acoustic velocity. 

All of the calculated acoustic velocities and Debye temperatures of diamond and stable XC_6_ and XC_12_ phases are shown in Table 3. Diamond is larger than C_6_ and doped structures in anisotropic and average acoustic velocity. The densities are increasing and the average acoustic velocities are decreasing with the atomic number, except NC_6_, which has a much smaller shear modulus. Compared to C_6_, the doping results in a decrease in the average acoustic velocity and Debye temperature. For the element S, which makes both XC_6_ and XC_12_ phases stable, the average acoustic velocity of SC_6_ decreases by 38.65% than C_6_, and that of SC_12_ by 35.96%. Furthermore, it can be found that the Debye temperature is decreasing with the atomic number, except SC_6_. The *Θ*_D_ characterizes the strength of the covalent bond in solids, so the strength of the covalent bond is lower for the phase which has the larger atomic number of doping atom. 

**Figure 8:**
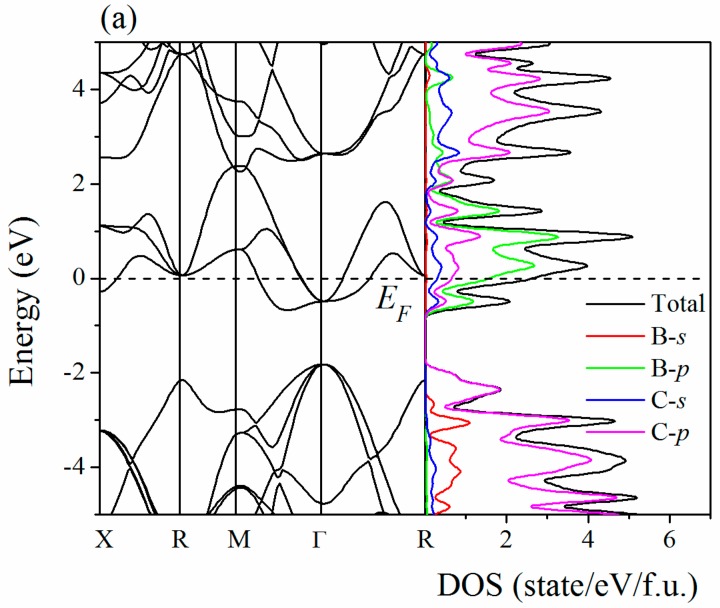

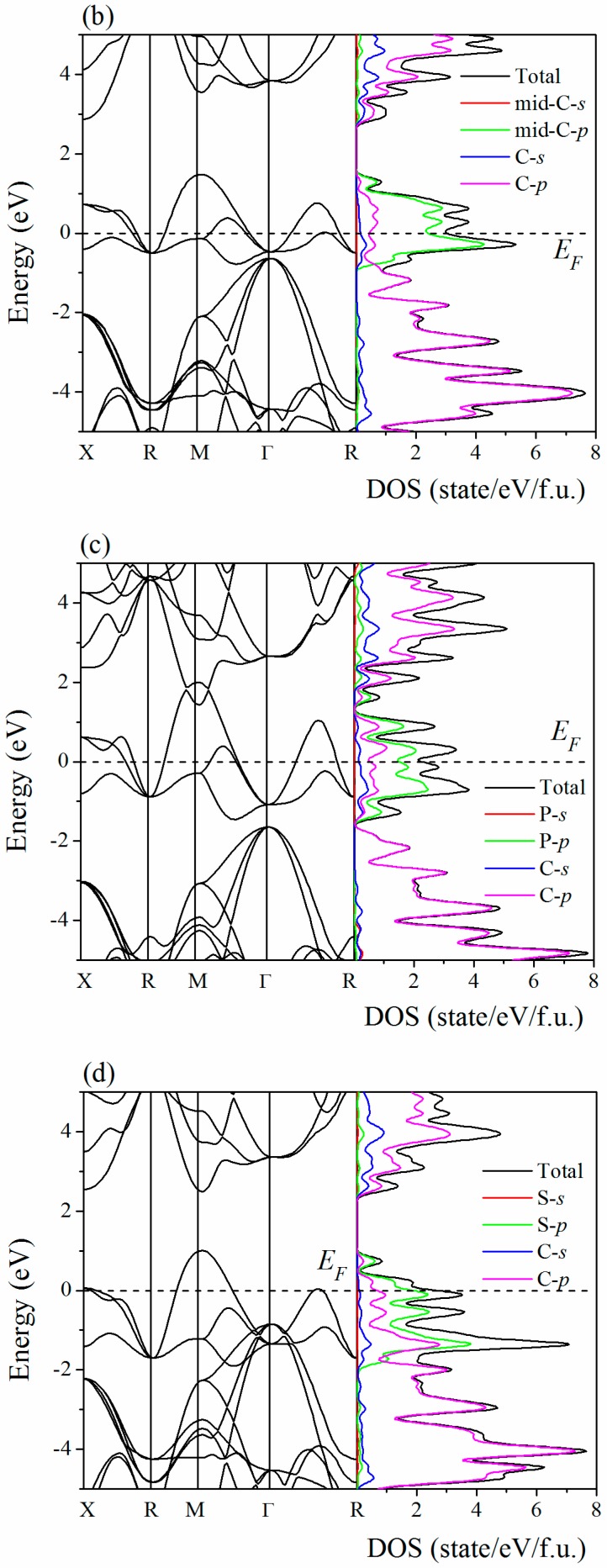

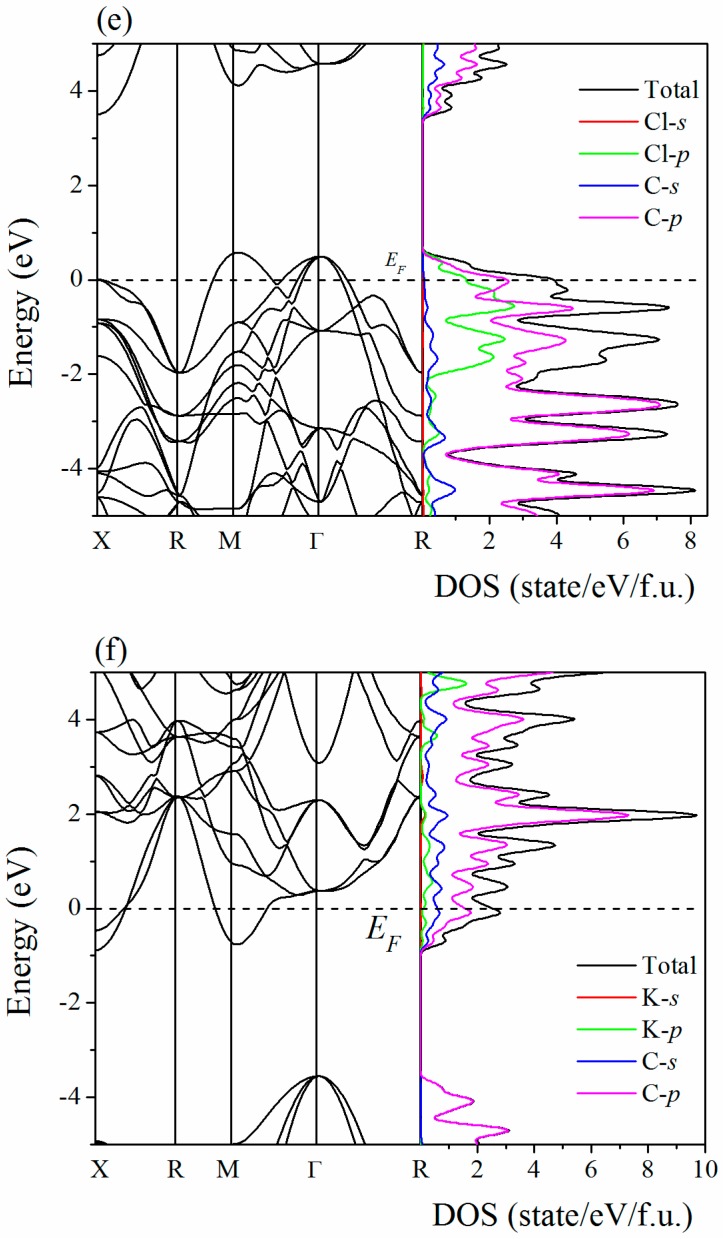
Electronic band structure and density of state of BC_12_ (**a**); CC_12_ (**b**); PC_12_ (**c**); SC_12_ (**d**); ClC_12_ (**e**); and KC_12_ (**f**).

Figure 8 shows the electronic band structure and density of state (DOS) of XC_12_ stable phases. The dash line represents the Fermi level (*E_F_*). The electronic properties of XC_6_ have been studied in [13]. For XC_12_, all of the band structures cross the Fermi level in the Brillouin zone, showing the metallic nature. The conduction band and valence band are mainly characterized by the contributions of C-*p* states, whereas the DOS near the Fermi level originated from the *p* orbital electrons of the doped element, except the ClC_12_ and KC_12_.

## 3. Computational Methods

The calculations are performed with the first-principles calculations. The structural optimizations are using the density functional theory (DFT) [31,32] with the generalized gradient approximation (GGA), which is parameterized by Perdew, Burke, and Ernzerrof (PBE) [33]. The Broyden-Fletcher-Goldfarb-Shanno (BFGS) minimization scheme [34] was used in the geometry optimization, and the total energy convergence tests are within 1 meV/atom. When the total energy is 5.0 × 10^−6^ eV/atom, the maximum ionic Hellmann-Feynman force is 0.01 eV/Å, the maximum stress is 0.02 GPa and the maximum ionic displacement is 5.0 × 10^−4^ Å, the structural relaxation will stop. The energy cutoff is 400 eV, and the K-points separation is 0.02 Å^−1^ in the Brillouin zone.

## 4. Conclusions

By using the first-principles calculations, the analyses on the mechanical properties of XC_6_ and the further exploration of XC_12_ structures are given. The formation enthalpies of dynamically stable XC_6_ phases and all of the XC_12_ structures, and the elastic constants, are calculated. There are ten structures which have the mechanical and dynamical stability (C_6_, HC_6_, NC_6_, SC_6_, BC_12_, CC_12_, PC_12_, SC_12_, ClC_12_, and KC_12_). The elastic modulus and anisotropy of the ten structures are studied and, in these structures, C_6_ has the lowest elastic anisotropy and the anisotropy increases with the atomic number. The doping leads to the weakening in mechanical properties and the increase in the elastic anisotropy. In addition, Debye temperatures and the anisotropy of acoustic velocities are also studied. The electronic properties studies show the metallic characteristic for XC_6_ and XC_12_ phases.

## Figures and Tables

**Table 1:** Calculated lattice parameter *a*, elastic constants *C*_ij_ (GPa), mechanical stability, bulk modulus *B* (GPa), shear modulus *G* (GPa), Young’s modulus *E* (GPa), Poisson’s ratio ν, and *B*/*G* ratio.

Materials	*a*	*C*_11_	*C*_12_	*C*_44_	Mechanical Stability	*B*	*G*	*E*	ν	*B/G*
Diamond	3.566 ^a^	1053 ^a^	120 ^a^	563 ^a^		431 ^a^	522 ^a^	1116 ^a^	0.07 ^a^	
C_6_	4.375	803	95	307	stable	331	325	735	0.13	1.018
	4.34 ^b^					352 ^b^				
HC_6_	4.390	607	215	344	stable	346	275	652	0.186	1.258
LiC_6_	4.491	634	118	−78	unstable					
NC_6_	4.446	414	295	162	stable	335	108	293	0.354	3.102
OC_6_	4.434	196	407	216	unstable					
FC_6_	4.427	269	370	335	unstable					
NaC_6_	4.566	659	91	−548	unstable					
AlC_6_	4.618	497	162	−59	unstable					
SiC_6_	4.614	527	165	−66	unstable					
PC_6_	4.605	542	179	−132	unstable					
SC_6_	4.608	683	115	90	stable	305	146	378	0.294	2.089
ClC_6_	4.613	92	374	104	unstable					
HC_12_	4.383	103	461	336	unstable					
LiC_12_	4.444	695	108	32	stable	304	93	253	0.361	3.269
BeC_12_	4.451	743	98	289	stable	313	302	686	0.135	1.036
BC_12_	4.439	684	136	233	stable	319	248	591	0.191	1.286
CC_12_	4.376	689	146	214	stable	327	235	569	0.21	1.391
NC_12_	4.415	275	361	278	unstable					
OC_12_	4.404	−661	830	526	unstable					
FC_12_	4.401	−33	529	476	unstable					
NaC_12_	4.476	741	77	−9	unstable					
MgC_12_	4.508	667	108	31	stable	294	89	240	0.363	3.303
AlC_12_	4.513	645	123	56	stable	297	110	294	0.335	2.700
SiC_12_	4.511	559	170	−25	unstable					
PC_12_	4.504	645	141	144	stable	309	181	454	0.255	1.707
SC_12_	4.502	397	273	251	stable	314	144	375	0.301	2.181
ClC_12_	4.503	349	297	295	stable	314	123	326	0.326	2.553
KC_12_	4.512	779	53	18	stable	295	93	252	0.357	3.172
CaC_12_	4.543	734	58	−2166	unstable					

^a^ Ref [14]; ^b^ Ref [12].

**Table 2:** Universal elastic anisotropy index (*A^U^*), Zener anisotropy index (*A*), and percentage anisotropy in shear (*A_G_*).

Parameter	C_6_	HC_6_	NC_6_	SC_6_	BC_12_	CC_12_	PC_12_	SC_12_	ClC_12_	KC_12_
*A^U^*	0.024	0.398	1.30	1.77	0.032	0.068	0.3814	2.752	11.084	21.252
*A*	0.8672	1.755	2.723	0.317	0.851	0.788	0.572	4.048	11.346	0.0496
*A_G_* (%)	0.243	3.752	11.567	15.016	0.315	0.678	3.714	21.596	53.098	68.612

**Table 3:** Density (g/cm^3^), anisotropic acoustic velocities (m/s) and average acoustic velocity (m/s).

Parameters		Diamond	C_6_	HC_6_	NC_6_	SC_6_	BC_12_	CC_12_	PC_12_	SC_12_	ClC_12_	KC_12_
ρ		3.517	2.857	2.869	3.252	3.535	2.941	2.992	3.182	3.206	3.265	3.313
[100]	*v_l_*	17,303	16,765	14,546	11,283	13,900	15,251	15,175	14,237	11,128	10,339	2331
	[010]*v_t_*_1_	12,652	10,366	10,950	7058	5046	8901	8457	6727	8848	9505	2331
	[001]*v_t_*_2_	12,652	10,366	10,950	7058	5046	8901	8457	6727	8848	9505	2331
[110]	*v_l_*	18,079	16,267	16,222	12,603	11,762	14,786	14,528	12,991	13,520	13,758	11,446
	$\left[1\bar{1}0\right]$*v_t_*_1_	11,517	11,131	8265	4277	8963	9652	9526	8899	4398	2822	10,467
	[001]*v_t_*_2_	12,652	10,366	10,950	7058	5046	8901	8457	6727	8848	9505	2331
[111]	*v_l_*	18,330	16,098	16,744	13,013	10,956	14,628	14,306	12,548	14,228	14,722	9813
	$\left[11\bar{2}\right]$*v_t_*_1,2_	11,907	10,882	9247	5367	7877	9409	9184	8239	6244	5952	8652
*v_l_*		17,901	16,356	15,761	12,136	11,889	14,851	14,629	13,151	12,563	12,100	11,246
*v_t_*		12,183	10,666	9791	5763	6427	9183	8863	7542	6702	6138	5298
*v_m_*		13,282	11,692	10,792	6483	7173	10,128	9795	8378	7487	6880	5963
*Θ*_D_		2219	1823	1766	1047	1118	1598	1551	1303	1165	1069	926
